# Direct analysis of volatile components from intact jujube by carbon fiber ionization mass spectrometry

**DOI:** 10.1186/s13065-019-0641-4

**Published:** 2019-10-31

**Authors:** Shihao Sun, Yihan Zhang, Peng Li, Hui Xi, Lei Wu, Jianxun Zhang, Guixin Peng, Yue Su

**Affiliations:** 1 Center for Chinese Medicine Therapy and Systems Biology, Shanghai University of Traditional Chinese Medicine, Shanghai, 201203 China; 2 Key Laboratory in Flavor & Fragrance Basic Research, Zhengzhou Tobacco Research Institute, China National Tobacco Corporation, Zhengzhou, 450001 China; 3 Technology Center, China Tobacco Henan Industrial Co., Ltd., Zhengzhou, 450001 China

**Keywords:** Jujube, Volatile, In situ analysis, Carbon fiber ionization mass spectrometry

## Abstract

In situ analysis of odor is an important approach to connect odor with chemical composition. However, it is difficult to conduct a rapid direct analysis of the odor sample because of low analyte concentration and sampling. To achieve the direct analysis, a carbon fiber ionization mass spectrometry (CFI-MS) method has been developed and applied to measure volatile components releasing from intact jujube. To build the CFI source, a 2.0-cm long carbon fiber bundle was integrated on the pin of a commercial corona discharge needle by mean of a 1.3-cm long stainless hollow tube. Odor sample driven by N_2_ gas can be directly introduced to the carbon fiber bundle to complete the ionization of analytes. Acetic acid, ethyl acetate, ethyl caproate, octyl acetate, and damascone present in jujube were selected to evaluate the performance of the CFI-MS method on quantitative analysis of the gaseous sample. Good lineary was obtained (R^2^ ≥ 0.9946) between 5.0 and 500.0 ng/L with limits of detection (LOD) ranging from 0.5 to 1.5 ng/L. Recoveries of five volatile compounds for the spiked jujube samples were between 94.36 and 106.74% with relative standard deviations (RSDs) less than 7.27% (n = 5). Jujube of different varieties can be distinguished by principal components analysis based on the analytical results of volatile compounds. The developed method demonstrated obvious advantages such as simplicity, high throughput, good sensitivity and wide range of applicability, which will be an alternative way for in situ analysis of the odor sample.
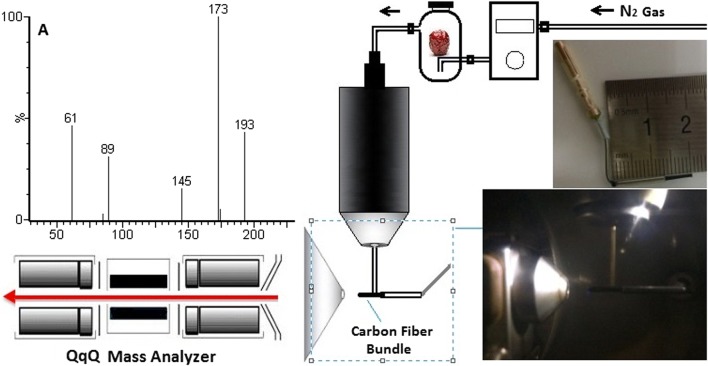

## Introduction

Jujube fruits (*Ziziphusjujuba* Mill.), which belong to the *Rhamnaceae* family, are indigenous to China for over 4000 years [1]. Jujube fruits not only have high nutritional and nutraceutical values but also present unique flavor which makes them very popular. The flavor of jujube is affected by a large number of factors, such as variety, growth stage, climate, storage and processing methods. Among all the factors, variety is one of the most important factors that affect the aroma of jujubes. And it is well known that odor recognition has been an important way to identify natural materials, such as natural flavor, traditional Chinese medicinal materials, and so on. However, this approach is empirical, time-consuming and laborious [2].

Odor is caused by volatile molecule acting on nasal receptors. According to the previous study, alcohols, acids, aldoketones, and esters were the main aroma compounds in different varieties jujubes [3]. Therefore, volatile components are usually employed to distinguish the odor characteristics of different samples. HS-SPME/GC–MS and E-nose are widely used for characterization volatile profile from different samples. However, limits of GC–MS such as multistep analytical procedures, poor sensitivity and time-consuming should be taken into account. Real-time analysis is a rapid, specific, and highly sensitive method. Thus, direct mass spectrometry techniques have drawn people’s attention to measure chemical constituents sustaining an odor feature and understand the link between chemical release and aroma perception [4, 5]. Direct mass spectrometry techniques, different from the methods involving chromatography, introduce volatile compounds continuously into mass spectrometry without sample preparation or chromatographic separation. Proton transfer techniques such as atmospheric pressure chemical ionization (APCI) [6–10], proton transfer reaction (PTR) [11–14], selected ion flow tube (SIFT) [15, 16], and ion-mobility (IM) techniques [17, 18] coupled to MS have applied to real-time analysis for the last 15 years.

Among the techniques mentioned above, APCI is recognized as one of the popular techniques for real-time analysis of volatile compounds [11, 19]. However, sample loss could cause by the injection method such as venturi effect [7, 20, 21] or the operation in the open air [22]. The loss of gas samples may lead to the degradation of sensitivity [23]. Several modifications have been made to introduce more ionized samples into the mass analyzer to reduce such sample loss. Jiang et al. used a larger inner diameter (0.53 mm i.d.) capillary to enhance the sample amount introduced into the ion source [24]. Cotte-Rodriguez et al. introduced the gas sample by a flexible tubing directly connected to the inlet of a mass spectrometer [25, 26]. Usmanov et al. directly introduced sample gas into an air-tight ion source pressed to the ion sweep cone of the mass spectrometer [23]. Although these methods have largely simplified analytical procedures and improved ionization capabilities, the highly efficient mass spectrometric direct quantitative analysis of low concentration and sensitive compounds in the gaseous sample still remains a large challenge.

Recently, Wu et al. proposed a new ambient ionization technique called CFI, which used a carbon fiber bundle as the ion source to assist in the ionization of small organics [27]. The carbon fiber bundle was placed close to the inlet of the mass spectrometer, in which an extra high voltage was indispensable to initiate corona discharge for the ionization of analytes. After that, Nahan et al. utilized a corona pin functionalized with a multi-walled carbon nanotube thread to achieve the carbon fiber ionization, in which the functionalized corona pin is capable of solid-phase microextraction of nonpolar analytes as well as ionization and sampling [28]. More recently, Wu et al. used single carbon fiber as the ion source to ionize analytes in vapor, liquid, and solid phases [29]. These proposed CFI methods are successful; however, the meticulous operation is required to care for the instrument setup including extra- high-voltage, functionalization of the corona discharge pin with its insert and seat, and the single carbon fiber in the methods. Although all of them declare to have a capability of quantitative analysis, quantitative data of targets in the gas sample was not presented in previous studies.

In order to facilitate the direct quantitative study of gaseous samples, we herein describe an improved CFI-MS method. A 2.0-cm long carbon fiber bundle was fixed on the commercial corona discharge needle by a stainless hollow tube. The carbon fiber bundle tip was placed close to the inlet of the mass spectrometer in axial. Analytes in the gaseous sample driven by N_2_ gas was directly injected on the carbon fiber bundle. The gaseous analytes were then ionized through interactions with the vaporized protonated water clusters [(H_2_O)_n_H^+^], which were generated by the CFI source. Subsequently, these analyte ions were transmitted to the mass spectrometer for analysis. Therefore, CFI-MS has been successfully applied for direct quantitative analysis of volatile components from intact jujube, which provides a simple and rapid analysis strategy for online analysis of the gaseous sample.

## Methodology

### Material and regents

Seven jujubes (*Ziziphusjujuba* Mill., moisture content of 20%), respectively originating from Xinjiang, Shandong, Hebei, Shanxi, Henan provinces, China, were purchased from Linze Agricultural trade company (Gansu Province, China). The Torayca^®^type T300B-3000 carbon fiber was provided by TORAY Industries, Inc.(Ehime, Japan). The 1.3 cm long stainless hollow tube (i.d. 1.0 mm) was tailored from the syringe needle (Shanghai high pigeon industry &trade co., LTD, China). Chemicals (99% purity) were provided by Adamas Reagent Co., Ltd (Basel, Switzerland), including acetic acid, ethyl acetate, ethyl caproate, and octyl acetate. Damascone (> 90% purity) from Sigma-aldrich; acetophenone-α,β-^13^C_2_ (> 99% purity) from Absin Bioscience Inc.(Shanghai, China); dimethyldichlorosilane, toluene and acetone from Sinopharm Chemical Reagent Co. Ltd (Shanghai, China); methanol (> 99% purity) from Merck (Darmstadt, Germany).

### Instrument

**Fig. 1:**
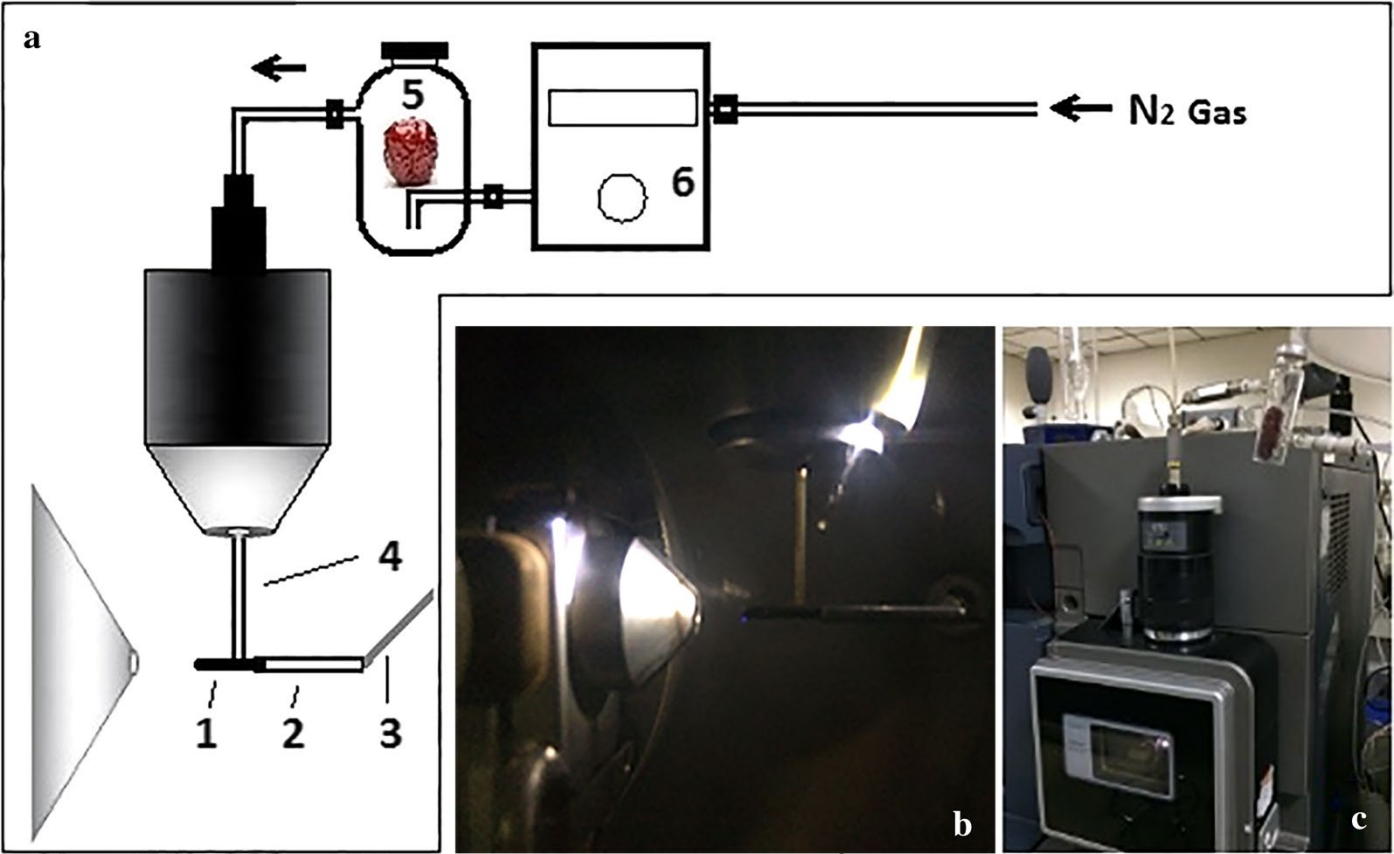
Schematic diagram of experimental setup. **a** Schematic diagram of analyzing volatiles from jujube by the improved CFI-MS: 1. carbon fiber bundle, 2. stainless hollow tube, 3. commercial corona discharge needle, 4. capillary, 5. sampling vessel, 6. mass flow controller; **b** photo of the improved CFI source; **c** photo of analyzing volatiles from jujube by CFI-MS

The experimental setup is illustrated in Fig. 1. A Xevo TQ-MS system (Waters, Milford, MA) was employed to perform MS experiments. The APCI ion source was modified to build our improved CFI source. A 2.0 cm long carbon fiber bundle passing through a stainless hollow tube (1.3 cm length, 1 mm i.d.) was ultrasonic-cleaned in methanol and dried by nitrogen blow. The tip of the corona discharge needle was carefully inserted into one side of the tube and made a 0.7-cm length carbon fiber bundle protrude from the other side of the tube. The carbon fiber bundle tip was placed 0.5 cm away from the inlet of the mass analyzer in the axial direction. The original APCI probe assembly has been modified as described in the Ref. [21, 24]. The original capillary (0.15 mm i.d., 0.39 mm o.d.) is replaced by a sampling capillary (0.53 mm i.d., 0.68 mm o.d., 30 cm length, methyl deactivated, SGE Analytical Science, Australia) which was installed in the instrument through a hole (0.78 mm i.d.) made by drilling the sample tube inlet fitting the APCI manifold and the APCI nozzle. One end of the sampling capillary was connected to a 20 mL sampling vessel while the other end was directly above the carbon fiber bundle in the vertical direction.

The homemade sampling vessel was made based on a commercial vessel (Agilent, USA) and the volume was measured by water. One inlet and one outlet were on the wall of the sampling vessel for gas transmission. The inlet tube extended to the bottom of the vessel and the outlet tube was on the top of the vessel. The volatile compounds in the vessel were swept along the nitrogen gas whose flow rate was controlled by a mass flow controller (MFC) and finally analyzed by improved CFI-MS. The sampling vessel was passivated according to the reported method [30]: the inner wall of the vessel was defatted with acetone and then dried for 2 h at 140 °C before usage; then the vessel was filled with a 5% (w/w) solution of dimethyldichlorosilane in toluene in order to deactivate the glass surface and the solution was removed after 24 h at room temperature; finally, the vessel was rinsed with toluene then methanol and dried in a stream of nitrogen [24].

### Sample preparation

The gaseous standard sample was achieved by vaporizing standard solutions in this study. Standard solutions were first prepared in methanol; then, 10 μL solution was spiked into the sampling vessel and fast sealed by a cap with Teflon-pad; After that, the gaseous sample reach equilibrium state and were introduced into ion source by N_2_. The concentration of analytes in the vessel was calculated by the ratio of addition amount to the volume of the vessel. The gaseous acetophenone-α,β-^13^C_2_ was also prepared by the method above and 50.0 ng/L was used as internal standard (Istd) in each sample. The gas in the vessel was directly introduced into the improved CFI-MS for qualitative and quantitative analysis.

Intact jujube and 10 μL of internal standard solution were added into the sampling vessel in turn. After fast sealing, the vessel was stored at ambient temperature. Then, the gas in the vessel was directly introduced into the CFI source for mass spectrum analysis.

### Mass spectrometry analysis

The sample vessel was placed between a MFC and CFI source. The volatile compounds in the vessel swept along the nitrogen gas whose flow rate was controlled by a MFC and injected to the CFI. The sampling process lasted for ~ 10 s. Then a vacant vessel replaced the sample vessel and the gas pipeline and the carbon fiber was cleaned by N_2_ gas flow. After a while, the MFC was closed and the vacant vessel was replaced by a new sampling vessel, and a new sampling process can be carried out.

The mass spectrometer was performed in positive-ion mode. MS operating conditions were as follows: discharge voltage: 3.0 kV; APCI probe temperature: 50 °C; desolvation gas flow: 80 L/h; cone voltage: 20 V. Tandem mass spectrometry experiments were based on collision-induced dissociation. Argon was used as collision gas at a flow rate of 0.15 mL/min.

### Method validation

The standard solutions of analytes were prepared with seven concentration levels, and a series of gaseous standard samples were ultimately obtained with the concentration ranging from 5.0 to 500.0 ng/L. A linear regression analysis with peak area ratio of analyte-to-Istd versus concentration of analyte was carried out to construct the calibration curves of volatile components. The LOD was defined as the concentration that yields a signal-to-noise ratio of 4. The limits of quantification (LOQ) was calculated to be the lowest analyte concentration that could be measured with a signal-to-noise ratio of 10.

The precision was determined by intra-day and inter-day variations. Standard solution with lower limit of quantification (LLOQ), low, middle, and high concentrations were prepared triplicate at each concentration. To obtain intra-day precision, one sample was analyzed three replicates at three different times 1 day (n = 9 at each concentration level). The RSDs of the five compounds were calculated. To obtain inter-day precision, one sample was analyzed three replicates at three consecutive days (n = 9 at each concentration level).

Recoveries at three levels of standard solutions were spiked into the sampling vessel with 2.0 g cutout jujube respectively, which were treated as unknown level samples for measurement. The recoveries (n = 5) of analytes were calculated as (total calculated amount-native amount)/spiked amount × 100%.

## Results and discussion

**Fig. 2:**
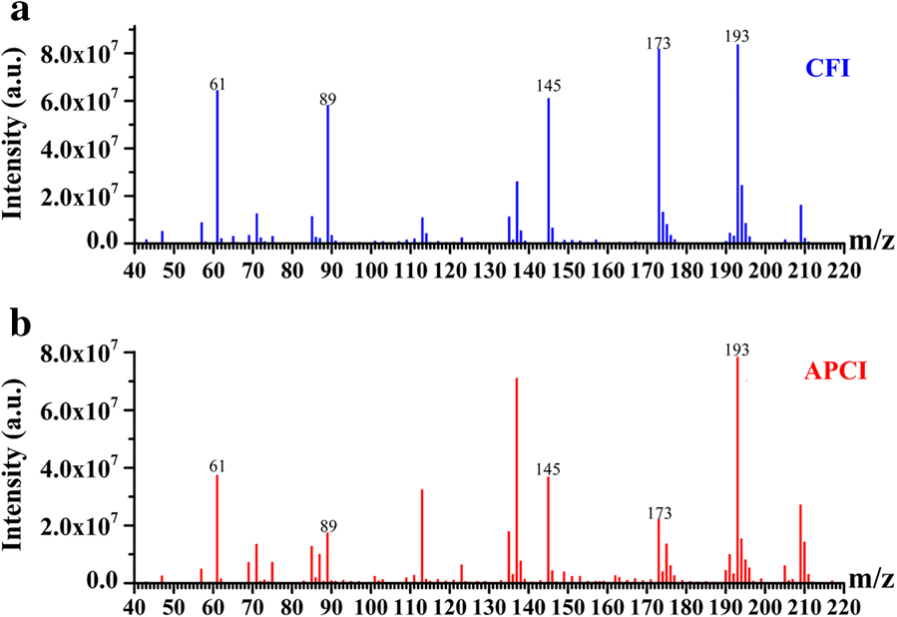
Full scan mass spectra of the standard gaseous sample by CFI-MS and APCI-MS: **a** CFI-MS; **b** APCI-MS

Initial experiments were performed to demonstrate the feasibility of the CFI-MS by analyzing the jujube sample (the result shown as Additional file 1: Fig. S1) and the standard gaseous sample consist of acetic acid, ethyl acetate, ethyl caproate, octyl acetate and β-damascone. The optimization of MS parameters was carried out to ensure optimal ionization conditions for analytes, using the MassLynx V4.1 software (Waters, USA). Although it is not necessary for desolvation gas and heating APCI probe for the CFI source, we were compelled to assign values to the two parameters due to the operating software. APCI probe temperature and desolvation gas flow were 150 °C and 80 L/h, respectively. The full scan mass spectrum of the standard gaseous sample was obtained by the CFI-MS as shown in Fig. 2a, and the signals at *m/z* 61, 89, 145, 173, and 193 were derived from acetic acid, ethyl acetate, ethyl caproate, octyl acetate, and damascone, respectively. Meanwhile, the sample was also analyzed by the APCI-MS, and the spectrum was shown in Fig. 2b. Comparing with the APCI-MS, fewer fragment ions were formed in the CFI-MS. Precursor ions [M+H]^+^ of analytes were the majority of signals in the spectrum. These results may suggest the CFI source is softer than the APCI sources.

### Parameters effecting on the CFI-MS method

The flow rate of the sample introduction into the ion source affects ionization efficiency. In our study, the CFI source was built based on a commercial APCI source: a carbon fiber bundle was integrated on the tip of the corona discharge needle, the APCI-probe was replaced by a sampling capillary, and the sample inlet of ion source was redesigned and connected to the sample vessel. Gaseous samples can be directly introduced into the CFI source driven by N_2_ gas. So the rate of introducing the sample is controlled by the flow of N_2_ gas. Meanwhile, a few analytes may remain on the carbon fiber and in the pipeline after sampling. A step to clean the ion source is indispensable to reduce memory effect. In order to obtain high ionization efficiency and high sensitivity, standard gaseous samples were employed to optimize the flow rate of N_2_ gas and the cleaning time of the ion source.

### Flow rate of N_2_ gas

**Fig. 3:**
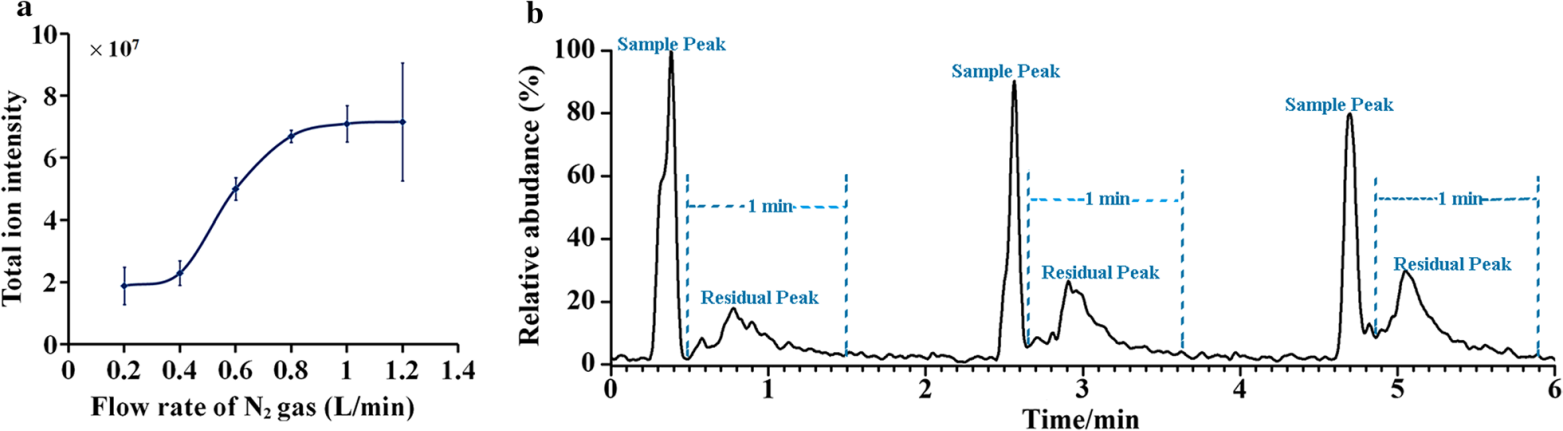
Optimizations of the CFI configuration: **a** flow rate of N_2_ gas used for the introduction of the sample vapor; **b** cleaning time for the removement of contaminant

With 50.0 ng/L of standard gaseous sample, a series of experiments were carried out at different flow rate of N_2_ gas (0.40, 0.60, 0.80, 1.00, 1.20 L/min), the results (deposited at Fig. 3a) indicated the increment of signal intensity as the flow rate of N_2_ increased. As a result, more volatile compounds can be introduced from the sampling vessel into the CFI source at the same time intervals. The results also showed that the total ion intensity was roughly constant with the flow of N_2_ gas over 0.80 L/min. When the flow rate of N_2_ gas was above 1.00 L/min, the carbon fiber tip started to swing and deviation of the total ion intensity became larger. Thus, the flow of N_2_ gas at 0.80 L/min was selected as the compromise proposal.

### Cleaning time

Further experiments were also performed to explore the effect of cleaning time. The sampling process was introduced in 2.4.1. We can observe from Fig. 3b that the sample peak immediately increased when the sample was spiked in the sampling vessel. When the vacant vessel was in the gas pipeline, a few analytes remained on the carbon fiber were also recorded by CFI-MS as the residuum peak shown in Fig. 3b. Thus, a cleaning step was required to reduce the memory effect of the device. In our study, N_2_ gas was used to clean the carbon fiber and the gas sample pipeline. We can infer from Fig. 3b that the residue can be removed completely within 1 min.

### CFI-MS method validation for quantification

Standard gaseous sample was used to validate the performance of the CFI-MS method. MS/MS data of analytes were obtained by the daughter scan mode of CFI-MS, including acetic acid, ethyl acetate, ethyl caproate, octyl acetate, damascene and Istd, which were shown in Additional file 1: Figs. S2–S7. MS/MS data of the ester compounds (showed in Additional file 1: Figs. S3–S5) indicated McLafferty rearrangement was an important way of analytes fragmentation in the CFI source.

Quantification was performed by multiple reaction monitoring (MRM) mode. The most abundant parent ion > daughter ion transition was selected to record the analytes at the optimal experimental conditions. Molecular ions, major fragment ions and optimized MRM parameters of targets and internal standard were summarized in Table 1.

**Table 1:** Molecular ions, major fragment ions and optimized MRM parameters for targets and internal standard

Analytes	Molecular ion	Collision energy (V)	Fragment ions (*m/z*)	MRM ion (*m/z*)
Acetic acid	61	20	43	61 > 43
Ethyl acetate	89	25	61, 29	89 > 61
Ethyl caproate	145	15	117, 89, 75	145 > 117
Octyl acetate	173	5	113, 71, 57	173 > 113
Damascone	193	20	137, 111, 69	193 > 137
Internal standard	123	25	45	123 > 45

**Fig. 4:**
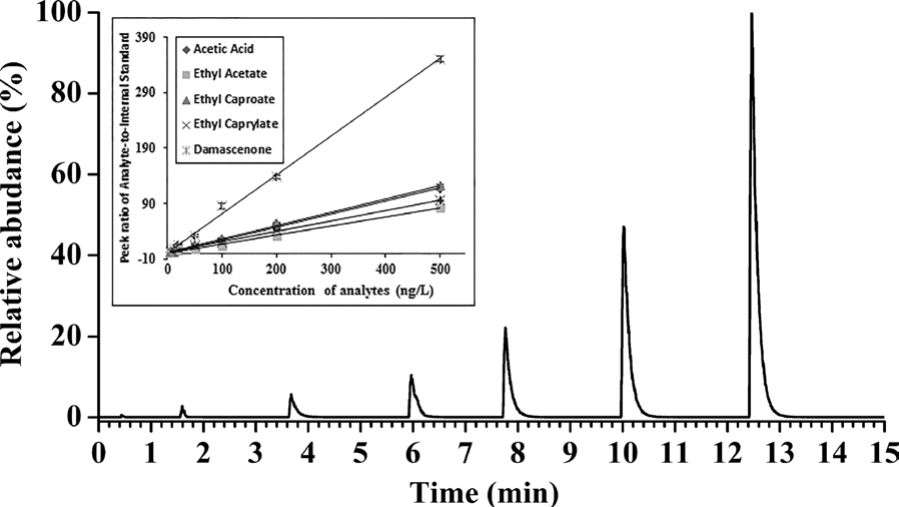
TIC spectrum of the standard gaseous samples by CFI-MS in the MRM mode and calibration curves of acetic acid, ethyl acetate, ethyl caproate, octylacetate, damascone were shown in the insert

Figure 4 showed the total ion chromatogram (TIC) spectrum of MRM for the standard gaseous samples. Calibration curve of each selected compound was constructed by the regression with the peak area ratio of analyte-to-Istd versus concentration of analyte, and the results were summarized in Table 2.

**Table 2:** Parameters for the performance of the CFI-MS method

Analytes	Calibration curves	R^2^	Linear range (ng/L)	LODs (ng/L)	LOQs (ng/L)	RSDs (%)
Acetic acid	Y = 0.2600X − 0.2740	0.9970	5.0–500.0	1.1	3.8	6.30
Ethyl acetate	Y = 0.1758X − 0.1701	0.9990	5.0–500.0	1.5	4.1	4.95
Ethyl caproate	Y = 0.2614X + 2.1391	0.9991	5.0–500.0	1.2	3.9	3.99
Octyl acetate	Y = 0.2102X + 2.2828	0.9992	5.0–500.0	0.5	1.8	6.44
Damascone	Y = 0.8143X + 1.5495	0.9946	5.0–500.0	1.2	4.0	5.45

A satisfactory correlation coefficient (R^2^) of 0.9946–0.9992 was achieved with the concentration ranging from 5.0 to 500.0 ng/L. The LODs and LOQs of analytes were in the range of 0.5–1.2 ng/L and 1.8–4.1 ng/L, respectively, which is on the same magnitude as HS–SPME–GC–MS and PTR-MS [31, 32]. The results of intra-day and inter-day precision were summarized in Additional file 1: Table S1, ranging from 2.63 to 8.53% for intra-day precision and 3.01% to 9.42% for inter-day precision, demonstrating good repeatability. Meanwhile, 10 μL of three level mixed standard solutions were spiked into three sampling vessels with Istd (acetophenone-α,β-^13^C_2_) and 2.0 g cutout jujube respectively, which were measured by the CFI-MS method. Calculated recoveries of all the analytes were between 94.36% and 106.74% with satisfactory RSDs less than 7.27% (detailed data summarized in Additional file 1: Table S2).

### CFI-MS analysis of volatile components of intact jujube

The mass range 40–300 amu was considered for data analysis after removing the background mass spectra. Additional file 1: Figs. S9–S15 showed the MS spectrum of volatile components from intact jujube by CFI-MS in the full scan mode. Masses *m/z* 61, 89, 103, 117, 131 appeared in the mass spectra in all samples. According to the previous studies [3, 33, 34], alcohols including 1-penten-3-ol, 2-ethyl-1-hexanol, demonstrated to the fruity and green aroma respectively, were shown as *m/z* 87 and 131 in all samples. Besides, *m/z* 89, 103, 117 were temporarily identified as butanoic acid, 3-methyl-butanoic acid, hexanoic acid corresponding to the cheesy flavor. However, the concentrations of these masses present little differences in the analytes.

The signals at *m/z* 61, 89, 145, 173, and 193 were assigned to acetic acid, ethyl acetate, ethyl caproate, octyl acetate, damascone by comparing with MS/MS data of standard substance respectively as shown in Additional file 1: Figs. S2–S6. Acetic acid, with a pungent, sour and overripe fruit flavor, was found in the headspace of all the seven samples in our studies. The relative amount of acetic acid was the highest among the acid components of the previous studies [35]. Esters, having a pleasantly fruity, floral aroma, are essential to the flavor of jujubes. The signals at *m/z* 117, 131, 159, 187, 201, 215, 229, 243, 257, and 271 were assigned to molecular ions ([M+H]^+^) of esters compounds according to the deduction of homologs. Moreover, ethyl caproate with sweet, fruity and pineapple-like aroma was identified in previous studies in different varieties of jujube [3, 31, 35]. These results showed ester compounds were the main chemical components in the headspace of jujube. We also found the signal intensity of damascone (*m/z* 193) was relatively high in sample A and D. Damascone, with fruity, floral, rose-like flavor, and low odor threshold value, may be an essential target to identify the variety of jujube samples.

**Fig. 5:**
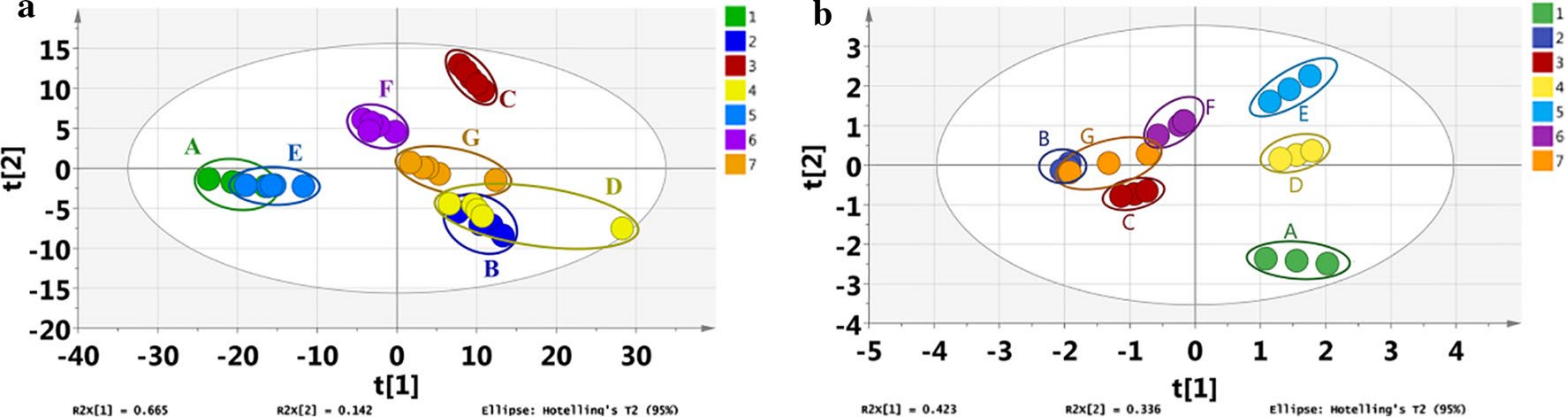
**a** Principle component analysis of the absolute intensities for all peaks (m/z of 45 to 299) of mass spectra of the headspace volatiles of seven jujube species from five provinces. Each point represents one sample; a total of 35 samples are included. **b** Principle component analysis of the quantitative results of acetic acid, ethyl acetate, ethyl caproate, octylacetate, damascone of the headspace volatiles of seven jujube samples. Each sample was measured three times. Detailed information of sample A to G: A. Jun Jujube (Akesu, Xinjiang); B. Yuan Jujunbe (Ningyang, Shandong); C. Hui Jujube (Qiangruo, Xinjiang); D. Po Jujube (Xingtang, Hebei); E. Goutou Jujube (Qingjian, Shanxi); F. Chang Jujube (Zaozhuang, Shandong); G. Yuan Jujube (Ningyang, Shandong)

Moreover, the discrimination species of the volatile statistics were demonstrated by the principle component analysis (PCA) without peak identification in Fig. 5a. PCA is used to check outliner and separate clusters when it’s hard to distinguish the differences among observation (i.e. jujube samples) by the experimental measurements (i.e., m/z from the mass spectra). In this study, PCA was performed on the air-subtracted mass spectral data to generate a graphical clustering of different jujube species. The first two principal components (PCs) accounted for 79.0% of the variance from all the variables (m/z values). Figure 5a showed a clear separation between the mass spectra of volatiles produced by jujube from different origins. The results obtained after PCA show that seven jujubes from five origins can be separated by their volatile data based on the origins.

Five selected compounds of jujubes from different origins were also quantified by the improved CFI-MS/MS. The quantitative results were summarized in Table 3 and the dataset of quantitative results was in a 2D PCA plot as shown in Fig. 5b. Obviously, the amount of the analytes was a significant difference between the jujube samples, and the content of some compounds (ethyl caproate, octyl acetate and damascone) in the jujube sample headspace were beyond their olfaction detection thresholds in air [36–41]. The first two principal components accounted for 75.9% of the total variance with concentrations of 42.3% and 33.6%, respectively. In general, the content difference of the compounds may be one reason resulting in an odor difference between jujubes from different origins. PCA results suggested that CFI-MS/MS can characterize jujube samples of different species based on the quantitative data of five selected volatiles from jujube.

**Table 3:** The concentration of compounds in the headspace gas from jujube samples

Sample Name	The concentration of analytes in the headspace (ng/L)^a^				
	Acetic acid	Ethyl acetate	Ethyl caproate	Octyl acetate	Damascone
A	137.79 ± 7.86	13.91 ± 1.44	142.48 ± 12.51	203.63 ± 20.54	48.97 ± 4.79
B	243.91 ± 7.29	5.68 ± 0.10	63.91 ± 4.20	77.43 ± 2.07	4.83 ± 0.36
C	151.94 ± 6.62	8.96 ± 0.76	276.62 ± 3.99	73.91 ± 5.77	6.46 ± 0.68
D	245.79 ± 11.33	18.04 ± 0.78	262.59 ± 19.38	182.36 ± 2.90	11.31 ± 1.13
E	390.82 ± 34.36	19.60 ± 0.92	185.66 ± 5.96	177.87 ± 10.14	4.37 ± 0.30
F	265.34 ± 19.67	17.92 ± 0.84	107.11 ± 8.55	93.74 ± 6.45	–
G	238.75 ± 18.11	7.80 ± 2.58	113.28 ± 20.64	98.99 ± 14.90	–

“–” means not detected

^a^Data are represented as the mean ± SD

## Conclusions

In this study, an improved CFI-MS method has been developed and applied to direct analysis of volatile compounds from intact jujube. The CFI source was built by integrating a 2.0 cm carbon fiber bundle on the commercial corona discharge needle. Gaseous sample was driven by N_2_ gas into the source and directly introduced the carbon fiber bundle to complete the ionization of analytes. Direct analysis of volatile components from intact jujube was achieved with good sensitivity, reliable quantification and high stability. Meanwhile, optimizations of two parameters were required, including the flow rate of N_2_ gas and the cleaning time of contaminant. This method demonstrated obvious advantages such as high throughput, good sensitivity, simplicity and a wide range of applicability. Therefore, the direct CFI-MS method was suitable for analysis of gaseous analytes at trace level and exhibited its potential value of researching the connection between chemicals release and perception.

## Supplementary information

**Additional file 1: Fig S1.** TIC spectrum of jujube sample by CFI-MS in the full scan mode. **Fig. S2.**–**Fig. S7.** MS/MS spectrum of acetic acid, ethyl acetate, ethyl caproate, octyl acetate, β-damascone, internal standard acetophenone-α,β-^13^C_2_. **Fig. S8.** TIC Spectrum of the standard gaseous samples sequentially analyzed by CFI-MS. **Fig. S9.**–**Fig. S15.** Mass spectra of different jujube samples. **Table S1.** Precision. **Table S2.** Data for the recovery of the CFI-MS method.

## Data Availability

Data and material are available on request.

## Abbreviations

CFI-MS: carbon fiber ionization mass spectrometry
RSDs: relative standard deviations
APCI: atmospheric pressure chemical ionization
PTR: proton transfer reaction
SIFT: selected ion flow tube
IM: ion-mobility
MFC: mass flow controller
Istd: internal standard
LOD: limits of detection
LOQ: limits of quantification
LLOQ: lower limits of quantification
MRM: multiple reaction monitoring
TIC: total ion chromatogram
