# Supplementary material for: Direct analysis of volatile components from intact jujube by carbon fiber ionization mass spectrometry
Source: BMC Chem. 2019 Oct 31;13(1):125. doi: 10.1186/s13065-019-0641-4 (PMC6823938; doi:10.1186/s13065-019-0641-4)
Supplement: Supplementary file 1 — Additional file 1: Fig S1. TIC spectrum of jujube sample by CFI-MS in the full scan mode. Fig. S2.–Fig. S7. MS/MS spectrum of acetic acid, ethyl acetate, ethyl caproate, octyl acetate, β-damascone, internal standard acetophenone-α,β-13C2. Fig. S8. TIC Spectrum of the standard gaseous samples sequentially analyzed by CFI-MS. Fig. S9.–Fig. S15. Mass spectra of different jujube samples. Table S1. Precision. Table S2. Data for the recovery of the CFI-MS method. [file 13065_2019_641_MOESM1_ESM.docx]

**Additional Information**

In order to study the feasibility of the CFI source, intact jujube samples were analyzed by CFI-MS, and each sample was analyzed continuously for two times. Fig. S-1 showed TIC spectrum of jujube sample by CFI-MS in the full scan mode.





**Fig. S1** TIC spectrum of jujube sample by CFI-MS in the full scan mode

The standard gaseous sample, consist of acetic acid, ethyl acetate, ethyl caproate, octyl acetate and damascone, was analyzed by CFI-MS in the daughter mode. MS/MS data of the compounds were showed as Fig. S2~S6. MS/MS spectrum of acetophenone-α,β-^13^C_2_ used as internal standard was also obtained.


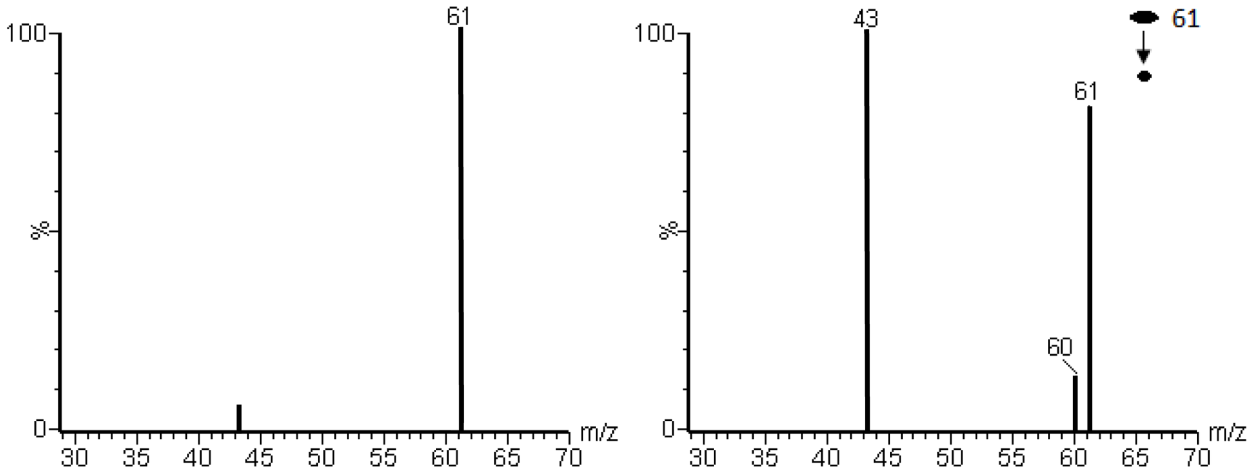


**Fig. S2** MS/MS spectrum of acetic acid


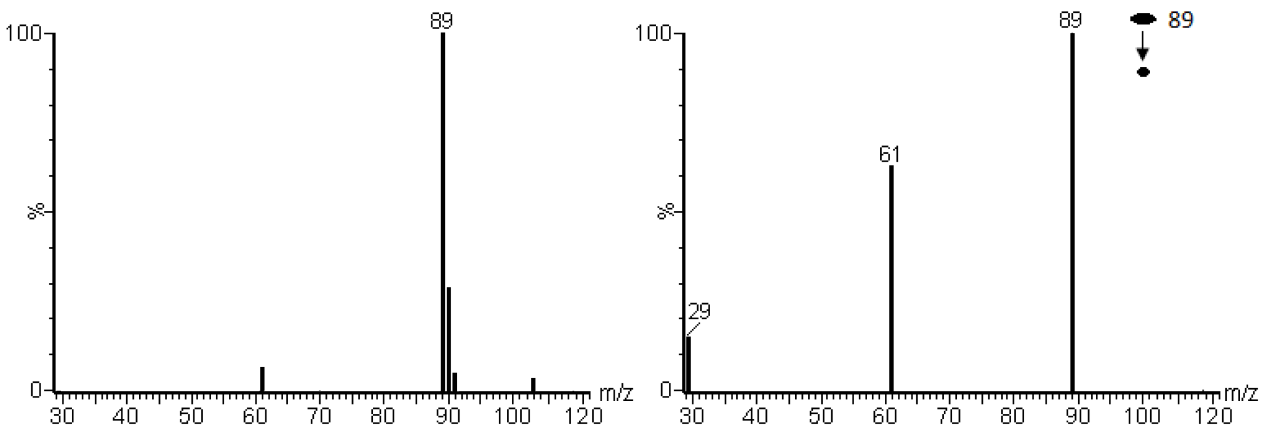


**Fig. S3** MS/MS spectrum of ethyl acetate


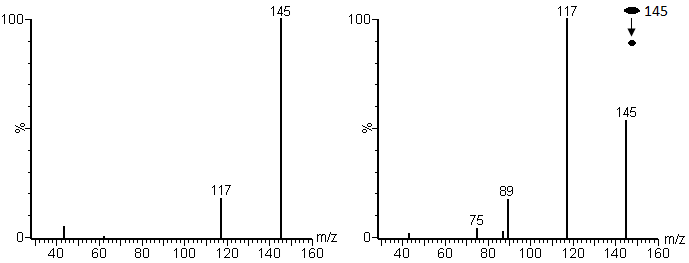
**Fig. S4** MS/MS spectrum of ethyl caproate


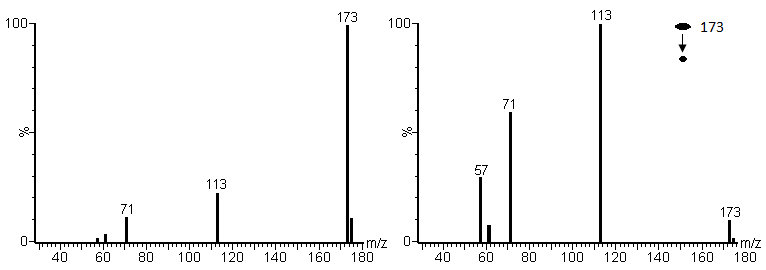


**Fig. S5** MS/MS spectrum of octyl acetate


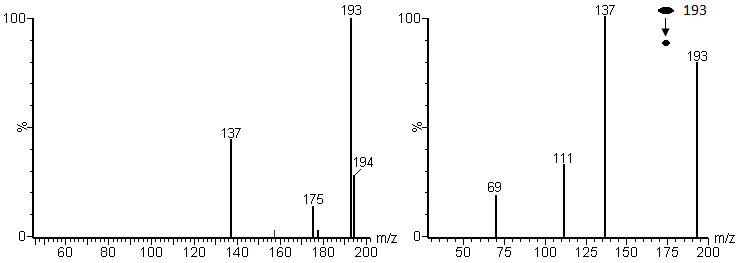


**Fig. S6** MS/MS spectrum of β-damascone


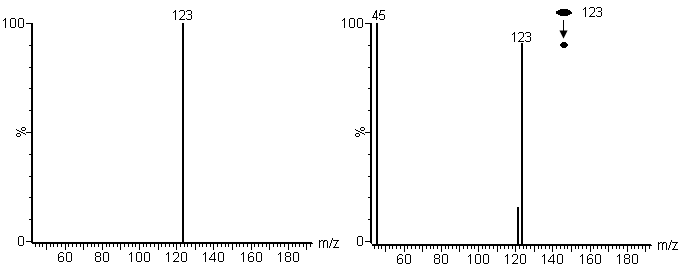


**Fig. S7** MS/MS spectrum of internal standard acetophenone-α,β-^13^C_2_

The standard gaseous sample with the concentration of 5 ng/L was sequentially analyzed by CFI-MS in the MRM mode. The results were showed as **Fig. S8**, which indicated the CFI-MS method maybe have a capacity of high throughput analysis with good repeatability.





**Fig. S8** TIC Spectrum of the standard gaseous samples sequentially analyzed by CFI-MS

Standard solution with lower limit of quantification (LLOQ), low, middle, and high concentrations (10, 100, 200 μg/L, 1000 μg/L) were prepared triplicate at each concentration. To obtain intra-day precision, one sample was analyzed three replicates at three different times one day (n=9 at each concentration level). The RSDs of the five compounds were calculated. To obtain inter-day precision, one sample was analyzed three replicates at three consecutive days (n=9 at each concentration level).

**Table S1** Precision (n=9)

| Analytes | Standard solution concentration (ng/L) | Precision (%) | |
| --- | --- | --- | --- |
|  |  | Intra-day | Inter-day |
| Acetic acid | 5 | 6.52 | 8.02 |
|  | 50 | 4.27 | 4.29 |
|  | 100 | 4.57 | 4.92 |
|  | 500 | 3.72 | 3.81 |
| Ethyl acetate | 5 | 8.25 | 8.95 |
|  | 50 | 3.57 | 3.72 |
|  | 100 | 2.63 | 3.01 |
|  | 500 | 3.04 | 3.17 |
| Ethyl caproate | 5 | 7.36 | 9.19 |
|  | 50 | 5.22 | 6.92 |
|  | 100 | 3.26 | 5.25 |
|  | 500 | 3.72 | 4.25 |
| Octyl acetate | 5 | 8.53 | 8.71 |
|  | 50 | 4.27 | 5.19 |
|  | 100 | 4.68 | 5.26 |
|  | 500 | 4.03 | 4.38 |
| Damascone | 5 | 7.05 | 9.42 |
|  | 50 | 3.05 | 4.25 |
|  | 100 | 3.51 | 3.71 |
|  | 500 | 3.29 | 3.85 |

The recovery of the CFI-MS method was obtained by measuring the 2.0 g cutout jujube samples spiked 10 μL of standard solution. 10 μL of three levels of standard solutions were respectively spiked into the sampling vessel with 2.0 g cutout jujube, which was treated as an unknown level sample for measurement. The recoveries (five replicate tests) of analytes were calculated as (total calculated amount－native amount) / spiked amount×100%. Recoveries of all the analytes were between 94.36% and 106.74% with relative standard deviations (RSD) in the range from 2.78% to 7.27%. Detail data were summarized as Table S2.

**Table S2** Data for the recovery of the CFI-MS method

| Anlyte | Added level (ng/L) | Mean±SD | Recovery (%) | RSD(%) |
| --- | --- | --- | --- | --- |
|  |  | (ng/L) | (n=5) | (n=5) |
| Acetic acid | 0 | 237.76±14.91 |  | 6.27 |
|  | 100 | 333.2±14.56 | 95.44 | 4.37 |
|  | 250 | 491.972±20.64 | 101.68 | 4.19 |
|  | 500 | 726.382±41.62 | 97.72 | 5.73 |
| Ethyl acetate | 0 | 10.46±0.47 |  | 4.49 |
|  | 5 | 15.216±1.11 | 95.12 | 7.27 |
|  | 15 | 24.656±1.59 | 94.64 | 6.45 |
|  | 50 | 62.052±3.57 | 103.18 | 5.75 |
| Ethyl caproate | 0 | 135.68±5.74 |  | 4.23 |
|  | 50 | 187.038±11.56 | 102.72 | 6.18 |
|  | 150 | 283.402±10.32 | 98.48 | 3.64 |
|  | 300 | 439.34±20.36 | 101.22 | 4.64 |
| Octyl acetate | 0 | 108.64±5.23 |  | 4.82 |
|  | 50 | 157.28±10.60 | 97.28 | 6.74 |
|  | 150 | 268.008±12.22 | 106.25 | 4.56 |
|  | 300 | 393.42±17.28 | 94.93 | 4.39 |
| Damascone | 0 | 12.07±0.61 |  | 5.05 |
|  | 5 | 16.788±1.12 | 94.36 | 6.64 |
|  | 15 | 26.268±1.46 | 94.65 | 5.56 |
|  | 50 | 65.4392±1.82 | 106.74 | 2.78 |


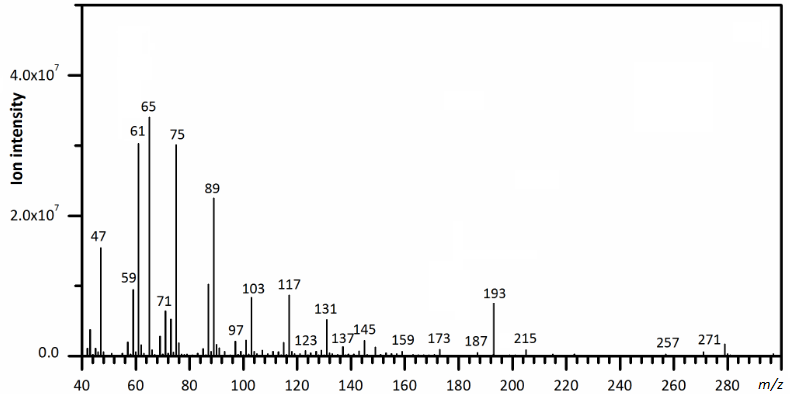


**Fig. S9 Akesu Jun Jujube, Xinjiang（A）**


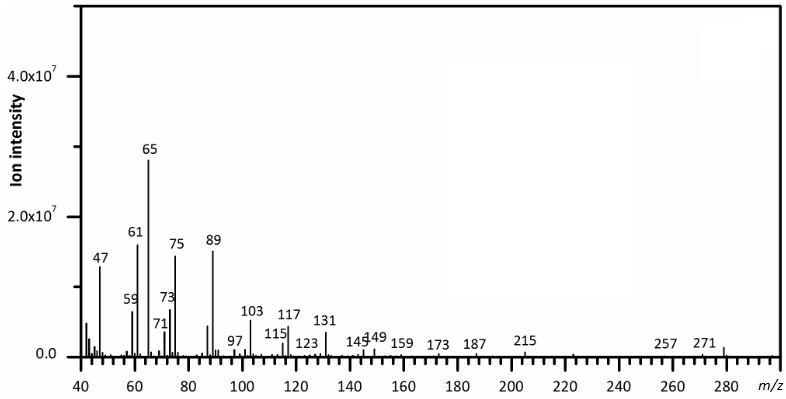


**Fig. S10 Ningyang Yuan Jujunbe, Shandong** **(B)**

**
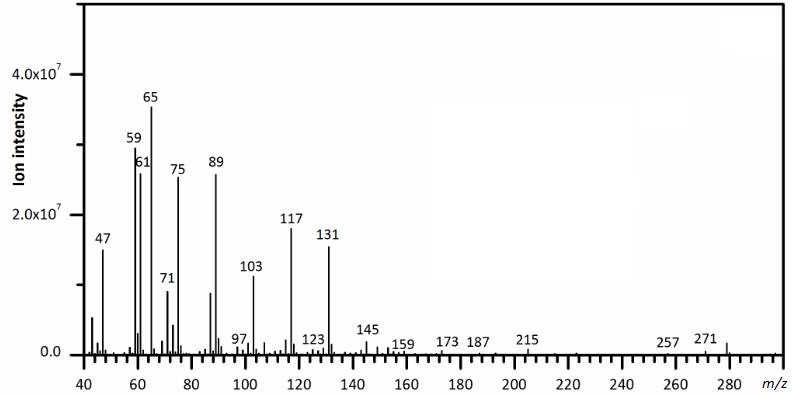
**

**Fig. S11 Qiangruo Hui Jujube, Xinjiang (C)**


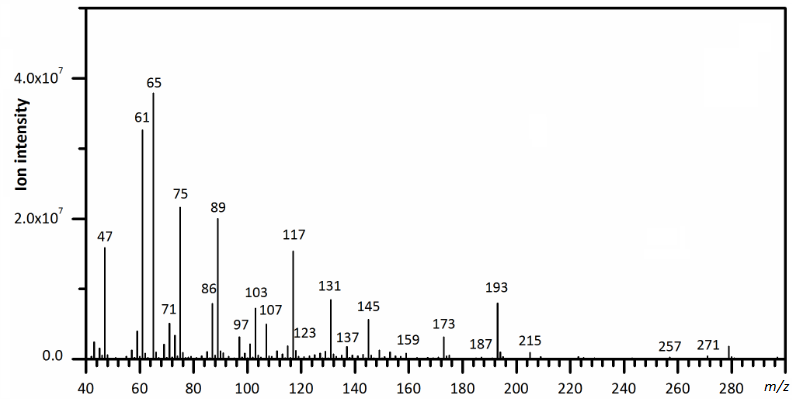


**Fig. S12 Xingtang Po Jujube, Hebei (D)**

**
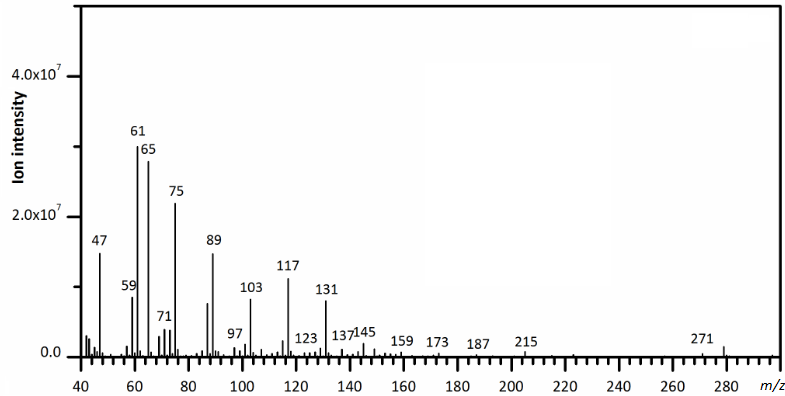
**

**Fig. S13 Qingjian Goutou Jujube, Shanxi (E)**


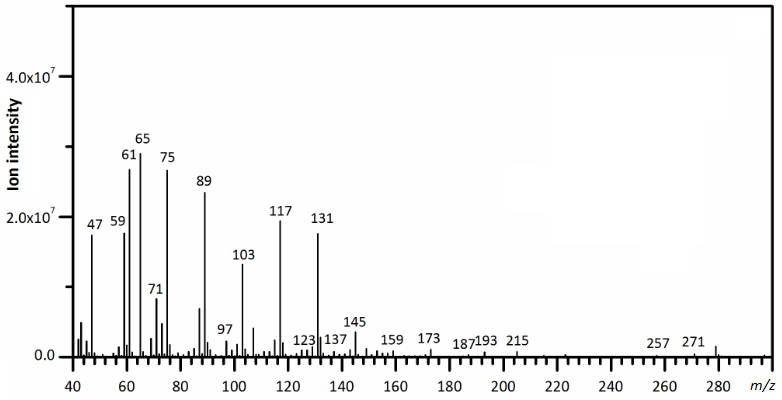


**Fig. S14 Zaozhuang Chang Jujube, Shandong (F)**


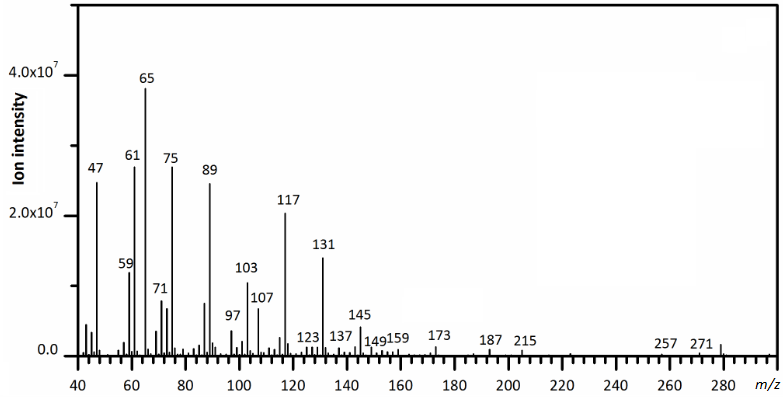


**Fig. S15 Ningyang Yuan Jujube, Shandong (G)**
